# Composition of Powdered Freeze-Dried Orange Juice Co-Product as Related to Glucose Absorption In Vitro

**DOI:** 10.3390/foods12061127

**Published:** 2023-03-07

**Authors:** María del Mar Camacho, Juan José Martínez-Lahuerta, Isabel Ustero, Eva García-Martínez, Nuria Martínez-Navarrete

**Affiliations:** 1 Food Investigation and Innovation Group, Food Technology Department, Universitat Politècnica de València, Camino de Vera s/n, 46022 Valencia, Spain; 2 CA Juan Llorens, Departamento Valencia-Hospital General, Consellería de Sanitat Universal i Salud Pública, Generalitat Valenciana, 46008 Valencia, Spain

**Keywords:** waste of orange juice, dietary fibre, enzyme inhibitor, glycaemic index, glycaemic load, glycaemia type 2 diabetes mellitus

## Abstract

The reuse of food by-products is crucial for the well-being of the planet. Considering the high content of nutrients and other bioactive compounds in many of them, investigating their suitability for use as human food ingredients is an interesting challenge. In this study, in addition to the proximate composition, phenol content and antioxidant activity (AOA = 3.2 mmol Trolox equivalent (TE)/100 g, db) of orange juice powder by-product (CoP), different in vitro properties related to carbohydrate metabolism have been characterised. Specifically, the glycaemic index (GI), the glycaemic load (GL), the glucose dialysis retardation index (GDRI = 13.6%), the glucose adsorption capacity (GAC = 22.5 mM) and the inhibition capacity of α-amylase (α-A = 46.9%) and α-glucosidase (α-G = 93.3%) of powdered orange juice waste have been determined and related to fibre and phenolics composition. Taking advantage of the high fibre content of the by-product (36.67%), its GL was calculated for a CoP dose that allows labelling the food to which it is added as a source of fibre. The low GI value (24.4%) and the low GL (0.918 g available carbohydrates per serving) allowed us to conclude that the product studied could be an interesting opportunity for the food industry to offer it as a healthy food ingredient to be included in the diet, especially for those suffering from type 2 diabetes mellitus. Of the total phenolic compounds (TP = 509 mg equivalent of gallic acid (GAE)/100 g, db), 68% were found in free fraction (FP), and their contribution to the total AOA was 40.6%, while this was 54.9% for the 32% of phenols bound to plant tissues (BP).

## 1. Introduction

One of the current interests directly affecting the agri-food industry is the use of the waste generated, following the circular economy model. Many food by-products are of high nutritional and potentially functional value [1,2]. Trying to combine both aspects, the processing of these by-products into food or ingredients for human consumption seems to be a highly interesting task. Focusing on the citrus sector, the juice-processing industry processes annually slightly more than 27% of the world’s orange production, which represents a number of about 28 million tons [3]. Considering that approximately 50% of the total weight of the used fruit becomes waste, it is interesting to find a way to take advantage of the bagasse, especially based on its high content of bioactive compounds, including dietary fibre and phenolics [4].

The presence of fibre in the orange peel is an important aspect to be considered since, in many countries, consumers’ fibre intake is insufficient and falls short of the 25 g/day recommendation of the expert committee of the Food and Agriculture Organisation of the United Nations (FAO) and the World Health Organisation (WHO) [5]. Many properties are attributed to fibre: regulating overweight and obesity, lowering the glycaemic index, preventing colon and rectal cancer, preventing cardiovascular diseases and combating constipation, among others [6]. Fibre mechanisms of action are based on the increase in chewing time and the sensation of satiety, delaying gastric emptying [7] and its ability to reduce serum glucose levels [8].

On the other hand, the current lifestyle of western society is rapidly and progressively deteriorating its eating habits by using highly processed products for its daily diet. As a consequence, there is an increase in certain metabolic pathologies that have, as one of their aetiological mechanisms or necessary collaborators, the ingestion of diets rich in carbohydrates and lipids. We are referring mainly to type 2 diabetes mellitus (T2DM) and obesity. In Spain, from 1987 to 2012, the age-adjusted prevalence of obesity increased from 8.0 to 16.5% and that of T2DM from 4.2 to 7.1%, particularly in men [9]. The International Diabetes Federation has released new figures showing that 537 million adults have diabetes worldwide, an increase of 16% (74 million) from previous estimates made by this Federation in 2019 [10]. The most recent data provided by the WHO indicate that in Europe, in 2022, overweight and obesity affected almost 60% of adults and one in three children, and T2DM affected 60 million people (approximately 9% of the adult population) [11]. Furthermore, and most worryingly, there is a shift in the incidence of cardiovascular pathologies associated with these diseases to earlier stages of life, many of them with fatal outcomes. In this sense, the design of food or food ingredients that help in the control of these pathologies related to postprandial hyper-glycaemic could promote the personalisation of diets for these population groups.

Two concepts are very important when it comes to choosing the right carbohydrate intake for our daily lives: GI and GL. The GI measures the ability of foods to raise glucose levels after ingestion compared to a reference food. In this sense, the GI is defined as the increase in the area under the glycaemic response curve resulting from the ingestion of 50 g of carbohydrates (CHO) of the tested food, expressed as a percentage of the response of the same amount of CHO from a standard food (glucose or white bread), taken by the same subject [12]. The value obtained for the reference food is 100, and that of the tested food is expressed as a percentage of this reference. In this way, foods with a high GI will cause rapid and significant upward fluctuations in blood sugar, while those with a low GI lead to smaller increases. In the daily diet, foods with a low GI (rice, potatoes, pasta, pulses, etc.) are the foods of choice, as the insulin requirement for their metabolism is lower. For the purposes of the GI, it is important to note that it depends not only on the composition of the food (type of starches, fibre content, type of CHO, fat content and acidity) but also on the techniques used for processing and cooking [13].

Despite the fact that GI values are useful to predict the glycaemic response of foods and to avoid unwanted postprandial hyperglycaemia, another concept that provides a more practical tool for understanding how much the ingestion of food will raise a person’s blood glucose level is the GL. It is calculated by multiplying the grams of total available carbohydrate (CHOa) per serving of food consumed by the food’s GI and dividing by 100 [14,15]. Thus, GL becomes a useful tool for helping people to account for both the quantity and the quality of carbohydrates present in a dose of an ingested food [15]. In a simplified way, we could say that while the GI refers to the rate with which a type of carbohydrate is absorbed and passes into the blood, the GL refers to the intensity of the insulin response that the food we have eaten will provoke. Both GI and GL are not only two very important indicators to consider for the population in general but especially for those suffering from diabetes or obesity. Knowledge of them enables us to choose the right carbohydrate intake for our daily lives and to assess the quantity of food that is likely to be suitable for maintaining an adequate blood glucose level [16,17]. For instance, some fruits are high in water and low in carbohydrates. In these cases, although their GI may be high, their GL will be low. Therefore, consuming one to two servings of these fruits will not raise blood glucose significantly when compared with other foods that have a high GI and GL.

In addition to controlling carbohydrate intake, another of the strategies to reduce postprandial hyperglycaemia is to limit the activity of digestive enzymes involved in carbohydrate metabolism in the intestinal tract. In this regard, α-amylase is the enzyme that degrades the polymeric substrate into shorter oligomers by catalysing the hydrolysis of α-1,4-glucan bonds present in starch, maltodextrins and other related carbohydrates; α-glucosidase catalysed the hydrolytic cleavage of disaccharides (maltose and sucrose) into monosaccharides (glucose and fructose) for absorption in the human intestine [18]. Inhibition of α-amylase and α-glucosidase enzymes is therefore important for the control of postprandial glycaemia. Inhibitors of the latter reversibly occupy the binding sites of α-glucosidase on sugar, thereby reducing the degradation of polysaccharides and delaying the intestinal absorption of carbohydrates, thus achieving their hypoglycaemic effect. Clinically, these inhibitors can be used to treat T2DM to prevent the onset of hyperglycaemia and other associated cardiovascular risk factors such as hyperlipemia and obesity [19].

With the aim of promoting the use of the by-product orange juice powder as a natural food ingredient, in addition to the proximal characterisation of the powdered orange juice co-product, in terms of water, sugars, fats, proteins, ash and dietary fibre content, both soluble and insoluble fractions, some properties related to carbohydrate metabolism have been analysed, these being the GI, GL, glucose dialysis delay index, glucose adsorption capacity and α-amylase and α-glucosidase inhibition capacity. These in vitro bioactive properties and their relationship with fibre and phenols are the most novel aspects of the study.

## 2. Materials and Methods

### 2.1. Obtaining the Powdered Orange Juice Co-Product

The orange juice waste used as raw material was provided by the Belles Arts Sant Carles cafeteria of the Universitat Poltècnica de València (Spain) in February 2022. To obtain the co-product powder (CoP), the residue, after completely removing the central column and seeds, was crushed and emulsified (Robot coupe blixer2, Valencia, Spain) and water was added at a ratio of 1/0.38 to facilitate the process and create a homogeneous mixture [20]. Emulsification was carried out for 5 min per 750 g of residue.

The mix was distributed into 16.8 cm radius aluminium plates to cover 1 cm thickness and frozen (Liebherr LGT 2325, Ochsenhausen, Germany) at −45 °C for at least 24 h until drying (Telstar LYOQUEST-55, Barcelona, Spain). The process conditions were −50 °C in the condenser, with a pressure of 0.05 mbar and a shelf temperature of 50 °C for 21 h. The freeze-dried cakes obtained were crushed (Thermomix^®^, Vorwerk, Spain) in batches of 40 g at 2000 rpm for 20 s, repeating the operation until all the powder obtained passed through a 200 µm sieve (CISA 200/50, Barcelona, Spain), with the help of a sieve shaker (RP 200 N CISA, Barcelona, Spain). The reason for doing so was to ensure the same solute composition in the powder as in the orange juice waste used as raw material.

### 2.2. Proximate Composition Analysis

The water content of the freeze-dried sample was determined with a Karl Fisher automatic titrator (Mettler Toledo, Compact Coulometric Titrator C10S, Worthington, OH, USA). Protein, ash, fat and total sugars content were analysed applying standard methods AOAC 955.04/90, 942.05/90, 920.39c and 31.042, respectively [21]. Total dietary fibre (TDF) content and its soluble (SDF) and insoluble (IDF) fractions were analysed by the enzymatic gravimetric method proposed by Johansson et al. [22] using a kit for the quantification of total dietary fibre (1.12979.0001, Sigma-Aldrich, Darmstadt, Germany). All analyses were performed in triplicate. Results are expressed as mean ± standard deviation.

### 2.3. Total Phenolic Compounds and Flavonoid Profile

To determine the TP of the CoP, both FP and BP were extracted and the sum was considered. The main flavonoids present in each fraction were analysed. In both cases, the method described by Camacho et al. [20] was used. Briefly, 1 g of sample was extracted with MeOH at 30 °C and the filtrate obtained was extracted again with the same solvent but at 60 °C. The sum of the two extracts was FP. The residue obtained from the extractions was then subjected to basic hydrolysis followed by acid hydrolysis and its filtrate was extracted with MeOH at 30 °C, yielding the BP extract. The quantitative analysis of total phenols in both extracts was carried out using a modified Folin–Ciocalteau spectrophotometric method, according to Alu’datt et al. [23]. Measurements were performed with a UV–Vis spectrophotometer (V-1200 VWR, VWR, Radnor, PA, USA) and the phenolic content was expressed as mg GAE/100 g dry basis (db), using a GAE standard curve (Sigma-Aldrich, Steinheim am Albuch, Germany).

The flavonoid profile of both FP and BP extracts was determined by UHPLC (Jasco equipment, Cremella, Italy) connected to a DAD detector (Jasco equipment, Cremella, Italy) and a Synergi 4 mHydro-RP 80 Å, LC column 150 × 4.6 mm (Phenomenex, Valencia, Spain) which was kept at 25 °C. The mobile phase used was composed of MeOH (A) and H_2_O (B), and linear gradient elution was performed starting at 30:70 (A:B) to reach 100:0 (A:B) at 30 min, with a flow rate of 1 mL/min and the injection volume was 10 μL. Chromatograms were recorded at 284 and 325 nm. The standard curves of the reference flavonoids, narirutin (Nat), hesperidin (Hes), didymin (Did), sinensetin (Sin), nobiletin (Nob) and tangerenin (Tan) (TCI Europe N.V., Paris, France) were used to quantify the flavonoids.

### 2.4. Antioxidant Activity

The AOA of the FP and BP extracts was carried out by the DPPH (1,1-diphenyl-2-pricrylhydrazyl) method [24]. Results were expressed in mmol TE/100 g (db) using a Trolox standard curve (Sigma-Aldrich, Steinheim am Albuch, Germany) and the same spectrophotometer described above.

### 2.5. α-Amylase and α-Glucosidase Inhibition Assay

Phenolic compounds have been described to play an important role in the inhibition of digestive enzymes [25,26,27]. In this case, a conventional phenolic compounds extraction was performed, according to the methodology described by Mccue et al. [28]. CoP (1 g) was mixed with 10 mL of distilled water, homogenized, centrifuged at 11,200× *g* at 4 °C for 20 min (Gyrozen 1236R, Daejeon, Republic of Korea) and filtered (0.45 μm membrane filter).

Inhibition of α-A was determined following the method described by Alu’datt et al. [23]. The mixture composed of 40 µL of extract or control (distilled water) with 400 µL of a starch solution (R05YI, ROQUETTE, Benifaió, Spain) and 200 µL of α-A (A3176-500U, Sigma-Aldrich, St. Louis, MO, USA) was kept at room temperature for 3 min and 3,5 dinitro salicylic acid (DNSA) method was used for detection of reducing sugars and determining the absorbance (A) at 540 nm. The percentage inhibition of the enzyme was calculated following Equation (1).

(1) $${\%\;\mathrm{Inhibition}}_{\propto -A}=\left(1\;-\;\left(\frac{A\mathrm{sample}}{A\mathrm{control}}\right)\right)\;\times \;100$$

Inhibition of α-G was determined using an α-G activity assay kit (MAK123, Sigma-Aldrich, St. Louis, MO, USA) following the protocol of the technical bulletin. 20 µL of phenolics extract was mixed with 200 µL of α-glucosidase solution and incubated at 37 °C for 20 min. The absorbance (A) was measured at 405 nm and the per cent enzyme inhibition was calculated following Equation (2).

(2) $${\%\;\mathrm{Inhibition}}_{\propto -G}=\left(100-\frac{A_{\mathrm{sample}}^{\mathrm{final}}{\;-\;A}_{\mathrm{sample}}^{\mathrm{initial}}}{A_{\mathrm{calibrator}}^{\mathrm{final}}{\;-\;A}_{\mathrm{Water}}^{\mathrm{final}}}\right)$$

### 2.6. Glucose Adsorption Capacity

The GAC of the CoP was performed according to Flores-Fernandez et al. [8]. CoP (1%) was added to 25 mL of a glucose solution (100 mM) in sextuplicate. Three of the mixtures were analysed for initial glucose using the glucose oxidase-peroxidase (GOD-POD) method for samples without resistant starch (Starch assay kit, STA-20, Sigma-Aldrich, St. Louis, MO, USA), using the reagents glucose oxidase-peroxidase (G3660), o-dianisidine dihydrochloride (D2679-1VL) and D-(+) glucose solution, all Sigma (Vidra-Foc, Barcelona, Spain) and measuring the absorbance at 540 nm. The remaining three mixtures were placed in an incubation chamber (Nüve Test Gabinet chamber TK120, Istanbul, Turkey) at 37 °C for 6 h, under constant agitation. Subsequently, they were centrifuged at 4000× *g* for 20 min and the final glucose in the supernatant was analysed in the same way as described above. GAC was calculated using Equation (3).

(3) $$\mathrm{GAC}=\frac{C_{i}-{\;C}_{f}}{m}\;\times \;V$$

where C_i_ and C_f_ are the glucose concentrations of the samples before and after incubation, respectively, V is the volume of solution and m is the weight of CoP used for the test.

### 2.7. Glucose Dialysis Retardation Index

The GDRI of the CoP was determined as described by Fuentes-Alventosa et al. [29]. Samples of 400 mg of sugar-free CoP (extracted twice with 80% ethanol) were completely hydrated with 15 mL of distilled water containing 30 mg of glucose. After 1 h under continuous agitation, the samples were transferred into pre-hydrated dialysis bags (15 cm length) (12,000 MWCO, Sigma Chemical Co, Merk, Darmstadt, Germany). Each bag and a control bag (with glucose, but without sample) were placed in a reservoir containing 400 mL of distilled water and kept in a thermostatic water bath at 37 °C for 1 h with constant agitation. After this time, the glucose concentration was determined spectrophotometrically (500 nm) by the GOD-POD method. GDRI from the dialysis bag into the dialysate was calculated using Equation (4).

(4) $$\mathrm{GDRI}=(100\;-\frac{C_{s}}{C_{c}})\;\times \;100$$

where C_s_ and C_c_ are the glucose concentrations of the samples and the control, respectively.

### 2.8. Estimated Glycaemic Index

An in vitro digestion of the CoP was performed as described by Brennan and Tudorica [30], involving a proteolytic stage followed by incubation with pancreatic α-amylase restricted by dialysis tubing. Every 15 min for 120 min, aliquots of 1 mL from the dialysate were withdrawn in triplicate for analysis of reducing sugar content using DNSA method. The withdrawn dialysate was replaced each time with sodium–potassium phosphate buffer. A standard curve using glucose was prepared. Equation (5) was used to calculate the hydrolysis index (HI), where A represents the “area under the glycaemic response curve (amount of glucose dialysed as a function of time)”, and Equation (6) for the estimated glycaemic index (GI_e_).

(5) $$\mathrm{HI}=(\frac{\mathrm{Asample}}{\mathrm{Acontrol}})\;\times \;100$$

(6) GIe=0.862 × HI+8.198

## 3. Results and Discussion

### 3.1. Characterization Proximal and Phytochemical Analysis

Table 1 shows the results obtained from the analysis of the proximate composition, the total content of free and bound phenols content and the sum of the identified flavonoids of the powdered co-product studied. The water content was in the range recommended by other authors for high-quality freeze-dried products [31]. Table 1 also shows that CoP is rich in nutritional ingredients such as total sugars, proteins and minerals. However, it also has a low-fat content. All these values were similar to those found by other authors on citrus peel [32,33,34].

The CoP may be considered a high-fibre food ingredient, with a total DF amount of 36.67 ± 0.11 g fibre per 100 g of co-product. These values are similar to those found in other studies, also for orange peel [35,36,37]. As regards the fibre fractions, SDF is responsible for an increase in viscosity in the intestine that hinders glucose diffusion and absorption, as well as α-amylase activity [8]. This enzyme is involved in the digestion and absorption of CHO by hydrolysing the α-1,4-glycosidic bonds within the glucose polymers ingested with the diet. IDF supports intestinal health by promoting regular bowel movements, delaying gastric emptying and possibly having a laxative effect [38]. In addition, IDF is able to bind carcinogens, mutagens and other toxic chemicals formed during food digestion, allowing their subsequent elimination through faeces [39]. Most foods containing fibre have more IDF than SDF, so in general, one-third of fibre is soluble, and two-thirds of fibre is insoluble [40]. In the case of CoP the IDF/SDF ratio (11.7) was significantly higher. Similar relationships have been found by other authors in previous citrus peel studies [35,36,37]. From this point of view, it does not appear that CoP can be proposed as an ingredient to assist in the control of postprandial glycaemia.

The content of TP present in the orange juice co-product powder was 509 ± 15 mg GAE/100 g co-product powder (db), of the same order as that found by Escobedo-Avellaneda et al. [41]. Of the total phenols analysed, 68% were found in the free fraction and 32% in the bound fraction, the latter associated with the cell wall and more difficult to extract (Table 1). The results obtained agree with those of Alu’datt et al., who showed that most phenols are found in free form in different fruits of the Rutaceae family [23].

**Figure 1:**
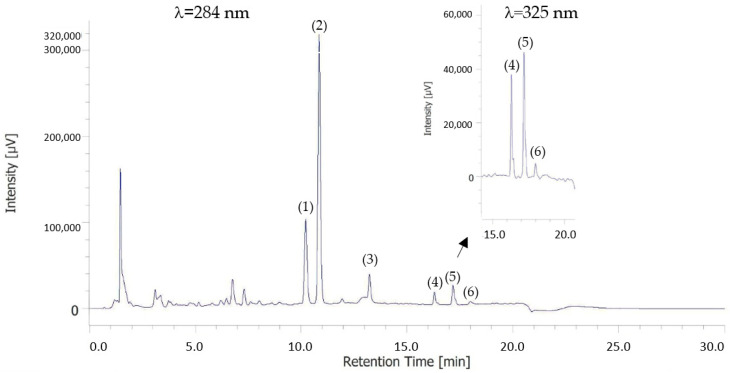
Example of chromatogram showing the flavonoid profile of the sample at 284 and 325 nm. (1) Naritutin. (2) Hesperidin. (3) Didymin. (4) Sinensetin. (5) Nobiletin. (6) Tangerenin.

Figure 1 shows an example of a UHPLC chromatogram which, at 284 and 325 nm, shows the peaks corresponding to the flavonoids identified in this study. Nat, Hes and Did, glycosylated flavanones, were identified at 284 nm. Sin, Nob and Tan, methoxylated flavones, were identified at 325 nm. The flavonoids identified in our study coincide with those found by Manthey and Grohmann [42] and Escobedo-Avellaneda et al. [41]. All the flavonoids eluted in the order indicated by other authors [43,44].

Table 1 shows the total flavonoid content quantified in the free and bound fractions. Specifically, the total content of Hes, Nat, Did, Sin, Nob and Tan associated with the powdered by-product (mg/100 g by-product) was 4045 ± 165, 529 ± 15, 128 ± 3, 69.5 ± 0.4, 74 ± 2 and 6.9 ± 1.5, respectively. Consistent with the references consulted, the main flavonoids found in each phenolic fraction were the flavanone glycosides Hes and Nat [41]. On the other hand, 96.4%, 99.5% and 90.8% of the analysed Hes, Nat and Did, respectively, and 100% of Sin, Nob and Tan were found in the free fractions.

To further evaluate the functionality of phenolic compounds, DPPH radical scavenging activity was determined, which is the most representative indicator reflecting the antioxidant activity of a plant extract [45]. The antioxidant activities of the free and bound extracts were 1.3 ± 0.2 and 1.9 ± 0.2 mmol Trolox equivalent (TE)/100 g (db), respectively. The total 3.2 mmol TE/100 g is in the range of other fruits recognised for their high antioxidant capacity [46]. It is worth noting that the bound fraction, with the lowest TP and identified flavonoid content, had the highest AOA. The distribution of antioxidant activity associated with each of the fractions indicates that the FF and BF fractions contributed 40.6% and 59.4% of the total antioxidant activity of orange peel, respectively. This is in concordance with Zou et al. [47] and Alu’datt et al. [23], who reported that bound phenolic compounds extracted from some citrus fruits had higher antioxidant activity than free phenolic compounds. Among the bound phenolic compounds, phenolic acids are the most abundant [48], and of these, cinnamic acids such as ferulic, coumaric, caffeic and synaptic acids, among others, have the highest antioxidant activity in citrus peel compared to other phenolic compounds [49]. In wild Chinese mandarins, ferulic acid is the main contributor to AOA [50]. Thus, it could be argued that these phenolic acids may be the major contributors of antioxidant activity to the BF fraction.

### 3.2. Bioactivity Assays

Both α-amylase and α-glucosidase are enzymes that help to release glucose, so their inhibition helps to lower glycaemia [51]. The activity of these enzymes seems to be inversely related to the presence of phenolic compounds in the sample due to their ability to interact with the enzyme to decrease its catalytic activity, either through conformational changes or by binding at the active site [25,26,27]. Xiong et al. [52] report that this inhibition is not only dependent on the concentration of phenolic compounds but also on their composition or phenolic profile. The effect on α-glucosidase and α-amylase of some compounds may be different. For example, in a study testing twenty-one naturally occurring flavonoids, hesperidin and kaempferol activated α-glucosidase and largely inhibited α-amylase, while luteolin and quercitrin largely inhibited both enzymes [53]. Rasouli et al. [54] also reported differential α-glucosidase/α-amylase inhibitory activities of phenolic compounds.

The α-A inhibition determined for CoP (Table 2) was higher than those obtained in other studies for different citrus powders acquired from the edible part of pummelo (30.2%), lemon (11.6%), grapefruit (18.9%) or different orange varieties such as shamouti (29.5%), clementine (29.8%) and red-orange (32.3%) [22]. The α-G inhibition values (Table 2) were higher than in the cases mentioned above, although similar to those of the lemon powder (100%). This may be due to the fact that the phenolic compound content of the edible part of the fruit is lower than that of the peel [55]. In any case, the ability of phenols to inhibit the activity of these two enzymes would indeed contribute to lowering blood glucose levels.

The GAC of the freeze-dried orange juice co-product (Table 2) was higher than that presented in other studies, such as that of citrus limmetta peel flour, which presented a value of 16.58 mM [8]. This high capacity could be related to the high content of insoluble fibre, which can effectively absorb glucose [56].

**Figure 2:**
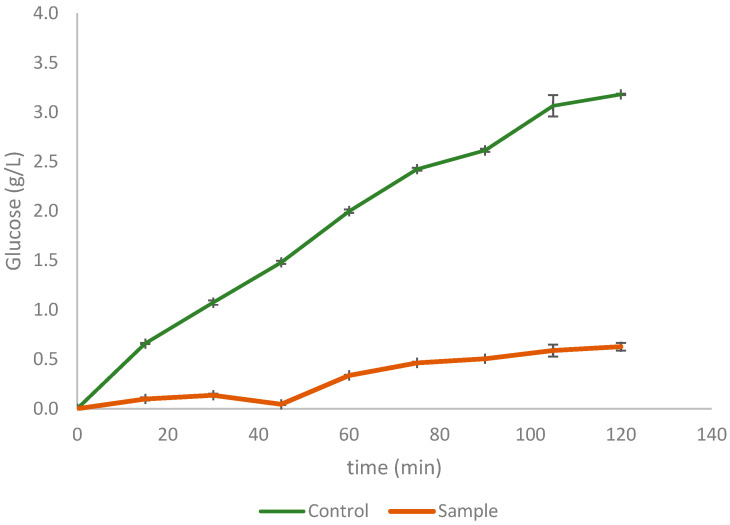
Glycaemic response curve of CoP versus glucose.

Figure 2 shows the glycaemic response curve of CoP versus control (glucose), used to calculate the hydrolysis index (Equation (5)), which is necessary for the estimation of the glycaemic index (Equation (6)), value shown in Table 2 for CoP.

As stated by Sivakamasundari et al. [13], foods with rapidly digestible, absorbed and metabolized carbohydrates are considered high GI (values with reference to glucose greater than or equal to 70). Medium GI foods are those with values greater than 55 and less than 70. Foods with carbohydrates whose physiological mechanisms are slower and have less impact on blood glucose and insulin levels are considered low GI (GI values less than or equal to 55). Carbohydrates with rapid absorption result in high GI values, while those with slow absorption produce flatter glycaemic responses and consequently low GI [57]. Considering these values, GIe of CoP is low, lower than those obtained for most fruits and other foods and more in the order of that of pulses [15,58], although similar to that of the peel of another citrus fruit such as grapefruit (19.89 ± 2.88) [59].

From the value of GI and the sugar content of the CoP, an estimate of its GL was made, by using the calculation procedure described in the introduction section. Assuming that the 46 g of total sugars analysed correspond to the CHOa of each 100 g CoP (Table 1, [60]), the GL calculated would be 11 g CHOa/100 g CoP. Although the GL may be expressed in this way, for convenience, it is usually referred to as a serving. As the powdered co-product studied is not intended for direct consumption but rather as an ingredient to be added in the preparation of different foods, it is difficult to propose the amount of CoP that a serving can contain. For reference, the following assumption was made. If this ingredient were to be added to a fibre-free food, such as yoghurt, e.g., in order to be labelled as a “source of” fibre, this would mean that the food would have to contain 3% fibre according to European legislation (Reg (EC) 1924/2006). As the fibre content of the CoP is 36.67 g fibre/100 g CoP (Table 1), 8.18 g CoP would need to be added to every 100 g of the formulated yoghurt to achieve the target. In this case, the GL of the CoP needed to guarantee the fibre content of a “source of fibre” food would be 0.918 g CHOa per serving. This is a very low GL, as a GL above 20 and average values between 11 and 19 are considered high and moderate glycaemic load values, respectively [15].

In this way, the CoP provides low available carbohydrates per serving (low GL), which are also slowly absorbed (low GI). Therefore, its consumption can be a tool to help control postprandial glucose levels, which is particularly suitable for people suffering from diseases such as T2DM or overweight.

Finally, with respect to IRDG (Table 2), the CoP presented a higher value than other citrus fruits such as lemon (5%) [61], but lower than other foods such as asparagus powder co-product [29], banana, dragon fruit and cantaloupe [62] or pea peel [63], among others, with values ranging from 15 to 48%. Despite the high fibre content of CoP, this low IRDG can be justified by the low soluble fibre content mentioned above (Table 1). Even so, CoP could be effective in slowing glucose absorption because of its high capacity for glucose adsorption and inhibition of the enzymes α-amylase and α-glucosidase.

## 4. Conclusions

According to the results obtained, the powdered orange juice by-product is a waste with a high content of insoluble fibre, which confers various beneficial properties for health, favouring a greater faecal volume and accelerating intestinal transit time. From this point of view, it can be recommended to be used as an ingredient to increase the fibre content of foods and come closer to WHO recommendations, while, due to its low glycaemic index and glycaemic load, it does not contribute to the increase of postprandial glycaemia. In addition, its high inhibition capacity, especially of α-glucosidase, but also of α-amylase, related to the high content of phenolic compounds, and its glucose adsorption capacity gives it a certain capacity for the regulation of postprandial glucose. In view of the above, its use seems particularly suitable to contribute to the personalization of the diet of people suffering from type 2 diabetes mellitus so that it helps them to increase their fibre intake and control blood glucose levels.

## Figures and Tables

**Table 1:** Water, total sugars, protein, ash, fat, fibre content (g/100 g sample) of powdered co-product. Total content of free and bound phenols (TP, mg GAE/100 g (db)) and the sum of the identified flavonoids (mg/100 g (db)) of the powdered co-product.

Water		3.57 ± 0.06
Total sugars		46.0 ± 1.17
Protein		4.38 ± 0.08
Ash		2.75 ± 0.02
Fat		0.79 ± 0.05
Fibre	Soluble	2.88 ± 0.05
	Insoluble	33.79 ± 0.11
TP	Free	346 ± 15
	Bound	163 ± 12
Sum of the identified flavonoids	Free	4690 ± 193
	Bound	158 ± 23

**Table 2:** Inhibition percentages of α-amylase and α-glucosidase, glucose adsorption capacity (GAC), estimated glycaemic index (GIe) and glycaemic retardation dialysis index (GRDI) of powdered co-product.

Inhibition α-amylase (%)	46.9 ± 0.6
Inhibition α-glucosidase (%)	93.3 ± 1.8
GAC (mM)	22.5 ± 1.3
GIe (%)	24.4 ± 0.7
GRDI (%)	13.6 ± 0.5

## Data Availability

The data presented in this study are available on request from the corresponding author.
